# Is the Realization of Reasonable Ideals in Dental Education near at Hand?

**Published:** 1903-07

**Authors:** Chas. C. Chittenden

**Affiliations:** Madison, Wis.


### IS THE REALIZATION OF REASONABLE IDEALS IN DENTAL EDUCATION NEAR AT HAND?[1]

BY CHAS. C. CHITTENDEN, D.D.S., MADISON, WIS.

REASONABLE ideals in dental education would seem to be defined by those standards as yet unattained and not yet in practical working, but which are admittedly possible and desirable in the estimation of the leading dental teachers, practitioners, and examiners of this and foreign countries, as expressed and promulgated by them personally and in the councils of the great national and international organizations. In no other country in the world is dentistry esteemed so truly a separate and distinct science and profession as in the United States of America.

Most of our schools are independent of and free from domination by the medical profession and medical schools, which is a condition not at all to be found in the schools of Europe, where our beloved profession is held as a mechanical art tacked on to a background of medical didactics.

Every enthusiastic lover of his profession probably has formed an ideal for himself, or for some one in whom he is interested, which should represent the summing up of his dream of educational perfection in dentistry, but the chief difficulty in the way of the general adoption of these ideals is that they are as various as they are ideal. One man will urge that an academic degree is of main importance, preparatory to the study of dentistry at all, while another will insist that beyond the simplest of common school preparation the wisest educational prerequisite for dental college is found in a term of pupilage in the office of a successful practitioner, where the study of text-books is a mere incident and the education is conducted on the principle carried out by the celebrated Mr. Squeers at Do-the-boys Hall, England; *i.e.*, teaching spelling as follows: "W-i-n, win, d-e-r, der, winder. Go and wash it."

And then there are all the intervening ideals that can be thought of, as wide in variation of detail and almost as numerous as the *personnel* composing the dental profession, and it will not be

---

[1] Abstract of a paper read before the American Medical Association, Section on Stomatology, New Orleans, May 5 to 8, 1903.

denied that there is fully as great a percentage of dreamers, cranks, and would-be prophets and leaders among us as is to be found in any other calling. It is out of the multitude of counsel, based on actual experience and effort, that reasonable standards can be evolved. The educators and examiners have been each and all striving for wise standards that will fill the educational ideals for some years to come, and also be practicable and attainable as a general fair minimum requirement by all educational institutions worthy the name. The dental literature for the past several years has overwhelmingly pointed to high school graduation and four years of special dental training as the minimum place where standards could be with safety set up and permanently established. The variations in standards for high schools in different portions of our country are great. Indeed, in some States having dental schools there is practically no established high school worthy the name. But, like all other commodities, schools accommodate their curriculum to the demand, and, at any rate, equivalents can be easily and fairly set up through the system of preliminary examination now in operation under the rules of the two National Associations of Faculties and Examiners, so that the decision as to what is a reasonable requirement in high school graduation can be very easily attained. And even where there are elective high school courses to be had, it is but a matter of detail to elect and maintain a reasonable equivalent. The college course has for several years been advanced to three years for graduation, and the addition of the fourth year to the curriculum, which is to be inaugurated in the present year in all our schools, completes what may be properly denominated reasonable ideals in dental educational standards.

If all the schools composing the National Association of Dental Faculties were departments of properly endowed universities, and thus relieved of the everlasting commercial handicap, there would be no question as to the immediate establishment of reasonable ideals. But the fact obtains that a large majority of them are practically struggling for existence and feel they cannot be too particular in their standards.

Having fairly determined what should be demanded and established in standards, the question arises, Who shall establish and be responsible for the carrying out of these standards? The answer is, The colleges, through their national associations, and the examiners, standing behind, protecting, insisting, and encouraging by the official authority that is in them vested. This being accepted as the proper method, all obstacles that beset the path and intercept the attainment of the goal must be squarely faced, with the view to most directly overcome them or pass around them in the onward course. The men to be convinced and enlisted and the means, the employment of which will most likely and directly conduce to the ends sought, are important. What is the power that must be invoked?

For over forty years dental schools have been pursuing their steady growth in size of classes and in numbers without there having been set up a single fixed minimum educational requirement for beginning a school course that could be in any sense claimed to be an established prerequisite. The demand for skilled practitioners was so great during the period of phenomenal growth and development of this great country following the close of the Civil War that perhaps it was as well that this should have been so, but to-day the demand is not so much for mere artisans as for educated, scientific, professional,—gentlemen, if you please,—who shall be competent to take the helm and guide the destinies of a great profession that is more nearly an exact science than any other, except it be perhaps the law.

Attempts at the raising of requirements have been continuous, and the past five years have seen the establishment of two years' high school preparation, and the present year inaugurates the four years' course.

The universities have been held back for years, hoping by slowly raising the educational requirements to enable the weaker schools to keep pace and so hold the national body together. It would seem that this has proved a mistaken policy, for the proceedings of the national association of colleges are filled with charges and trials, convictions and fines, against school after school for the violation of the plain rules of the body as regards the receiving and giving standing to students taken into their classes, with no other earthly object apparent than to swell the income of the struggling institutions. This is, of course, human nature, but it is none the less scandalous, notorious, and disreputable in the highest degree, and should be branded with the condemnation of the profession at large.

A case in point of what is constantly going forward in the effort to swell classes at whatever risk (and the half of which is never brought to light) is that of an endowed university which was compelled to refuse admittance to its classes the present year to about thirty matriculants because they did not possess the prerequisite education for admission to the third year of high school, as required by the rules of the two National Associations. These men applied elsewhere, and most of them, it is believed, are now enrolled in the classes of other schools belonging to the Association.

Another instance is where one school sought (and did in some degree succeed in its undertaking) to swell its own classes by sending out emissaries, cappers, or steerers, offering in wholesale lots to almost the entire classes of a competing school reduced tuition rates varying from $25 to $65 for the $115 advertised tuition fees.

Another instance of flagrant attempts on the part of schools in the full membership in the National Association of Dental Faculties to evade the rules is where one of them, by the admission of its dean, has taken into its Freshman Class for regular work a number of matriculants who had signally failed to pass the preliminary examination (set up by the National Association as its minimum standard for admission to classes) before the duly accredited appointee of the State Superintendent of Public Instruction, and then attempting to build up some sort of excuse for its conduct by not only attacking the educational fitness of the examiner,—who, by the way, is the honored principal of a district school in a large and flourishing Northern city,—but also by attacking publicly his character as an honest man and public servant, and thus attempting to excuse itself for having these men in its classes at all, even under the name of "specials" and "in our preparatory classes, besides doing regular freshman work." And this school is evidently banking on being able to control enough "inflooence" in the National Association to cover up or else explain away its conduct, or, at the most, escape with a "fine," as though payment of money could in any sense atone for the violation of the law of common honesty.

There is also the lamentable spectacle of schools posing as the very "ideal" of everything desirable, and whose official heads are shocked and hurt at the mere suggestion that any one should presume to criticise them or question their reputability, that are constantly and persistently flooding the secular press of the country with paid pictorial advertisements of their classes, foot-ball teams, handsome, noted, and extra-skilled teachers and instructors, exploiting the peculiar advantages of the institution and its professors over all competitors,—all done in a style to put to the blush the common advertisers of the cheap "dental parlors" type.

Such things unchallenged and unrebuked certainly do not tend to the realization of ideals, and most certainly do not offer incentives to enter our profession to the class of young men our profession most lacks. This is the sort of thing which has always stood in the way of the proper establishment and maintenance of ideal standards.

It is futile for this body, composed of the membership of the "reputable" colleges of this country, to adopt for the government and conduct of its members certain rules and standards and methods of procedure based upon high ideals of ethics and mutual protection, to be accepted by the profession at large as a criterion of the schools' reputability, and then devote a large part of its annual sessions to inventing excuses and apologies for flagrant and open violation of the letter or spirit, or both, of these standards and rules, thus giving to the outside world the very reasonable impression that the body is dominated by anything but the real spirit of building up ideals that their rules and standards would indicate. It is high time that the College Association awaken to a few realities of the situation. There is at this time, as a result of years of struggle and contention between schools and the examiners for the establishment of specific standards, accepted by the schools and made by the boards their measure of reputability,—there is now I say, a period of mutual accord and trust and peace between these bodies, which should be the harbinger of better things to come. The schools are assured of protection by the boards from any infringement and violation by competing schools, made and provided that the College Association will purge itself of the violators and enforce its own rules without fear or favor.

In order that ideals may be sooner and more surely established and maintained the boards of examiners, with all fairness and reasonable consideration for the interests of honest educational efforts, must nevertheless be mindful of their official oath to carry out not only the letter, but also the spirit and intent of the law for the protection of the public. They must in the National Association stand squarely together on reasonable requirements, and, more than all else, have the courage of their convictions in passing on the character of the colleges and their output, constantly having in mind that the goddess Justice does not close one eye and blink the other when on duty.

If the schools will rise to the occasion and set the pace, the examiners will most assuredly help and sustain them in holding it steadily up. If the National Association of Dental Faculties will not purge itself and be honest in enforcing its rules, there will be but one way to move the profession forward,—to wit, the better and honest schools will naturally unite themselves together, and the examiners must join them in a newer and higher emprise on a surer and it is to be hoped better-laid foundation.

